# Territorial Pattern Evolution and Its Comprehensive Carrying Capacity Evaluation in the Coastal Area of Beibu Gulf, China

**DOI:** 10.3390/ijerph191710469

**Published:** 2022-08-23

**Authors:** Menglin Ou, Xiaochun Lai, Jian Gong

**Affiliations:** 1 School of Public Administration, China University of Geosciences, Wuhan 430074, China; 2 School of Foreign Languages, China University of Geosciences, Wuhan 430074, China

**Keywords:** territorial pattern evolution, comprehensive carrying capacity, coastal area, the Beibu Gulf

## Abstract

Changes in the territorial pattern of the Beibu Gulf, an environmentally sensitive and ecologically fragile area in China, will directly or indirectly affect the regional ecological environment, while profoundly influencing economic development and human well-being. Therefore, it is significant to understand the ecological response in the process of territorial space changes in the Beibu Gulf to promote the coordination between sea and land and sustainable regional development. This paper used remote sensing image interpretation to generate land-use maps in 2000, 2010 and 2020, and then analyzed the spatial and temporal evolution of the territorial pattern of the Beibu Gulf from 2000 to 2020. Finally, this paper proposed a comprehensive carrying capacity evaluation system and explored the spatial functional zones of the coastal areas of the Beibu Gulf. The results showed that the demand for urban development and ecological protection between 2000 and 2020 increased built-up land and forestland by 386.71% and 25.56%, respectively, and reduced farmland by 28.33%. There was significant spatial heterogeneity in various land-use types. Where forestland is mainly distributed in the west, farmland is mainly distributed in the east, wetland is mainly distributed in the south, and orchards are spread throughout the whole area. The evaluation results of land resources, water resources and ecological conditions in the Beibu Gulf area showed that its comprehensive carrying capacity was high in the south and low in the north, and high in the west and low in the east. On this basis, this paper considered the actual situation of natural resources, ecological conditions, socio-economic development, protection and development in coastal areas; divided the study area into four categories: developed areas, priority development areas, ecological reserve areas and coastal reserve areas; and put forward corresponding control suggestions. The results of this paper could provide a scientific basis for regional development and territorial spatial planning in the coastal areas.

## 1. Introduction

The regional territorial pattern and ecological environment have changed significantly due to intense human activities, and the territorial pattern degree is higher in developing countries than in other regions [1]. As a land–ocean interaction area, the Beibu Gulf is an environmentally sensitive and ecologically fragile area, with frequent human activities and strong disturbances [2,3]. The Beibu Gulf Economic Zone was established in 2006, and it effectively promoted economic development [4]. Urbanization expansion and infrastructure construction is ongoing in this region. Rapid population growth and excessive resource consumption have destroyed the regional ecosystems and exacerbated environmental pollution [5]. Environmental problems such as seawater intrusion, vegetation degradation and land desertification have seriously affected the structure and function of territorial spaces and the regional sustainable development [6]. In the past decade, the Beibu Gulf has experienced both land and marine economic development [7], urban expansion [8], ecological restoration [9] and other human activities. Therefore, systematic analysis of territorial pattern is helpful for understanding the changes in land and sea ecosystems. Meanwhile, the identification of territorial characteristics and the land-use changes in coastal areas are of great significance for promoting the overall land and sea development.

Territorial pattern changes would directly or indirectly affect the regional ecological environment, and profoundly affect economic development and human well-being at the same time [10,11]. After the Chinese government launched ecological restoration projects such as returning farmland to forests, the Beibu Gulf responded positively, and ecological construction and ecosystem restoration received attention [12,13]. Ecosystems can provide human beings with a variety of services such as food, clothing, housing and transportation. They are basic support for human survival and development and play a vital role in maintaining material circulation and ensuring energy transmission [14]. At present, the temporal and spatial variations in coastal land use and ecological environment in the Beibu Gulf have been analyzed in some studies [5,15], but the significance is still unclear. It is an important way to predict the future development of the territorial space for assessing the territorial pattern changes [16]. Therefore, it is necessary to study the territorial pattern changes in this area to understand the ecological environment response in the process of territorial space changes, and this is of great significance for promoting land–sea coordination and regional sustainable development.

Currently, the gaps between environmental protection and social economic development could affect the sustainable development [17]. The world is faced with problems such as resource shortage and environmental pollution, which threaten economic and social development [18]. Chinese coastal areas have a large population and a developed economy, but they are facing increasing constraints on resources and the environment because the relationship between the environmental protection and social economic development is not well coordinated [19]. Driven by the trend of coastalization, there are many Binhai New District and New City projects under construction and planned in the Beibu Gulf [20,21]. These plans and constructions reflect the lack of regional integration in the utilization of coastlines and land resources in each district. The current development boom is likely to cause disorderly development of valuable, non-renewable resources and damage to coastal public resources [13,22]. How to use and protect the existing coastal zone resources is key for planning the coastal zone of the Beibu Gulf, understanding the ecological function of the coastal zone, learning the industrial advantages of the Beibu Gulf Economic Zone, promoting the sustainable use of coastal zone resources, and realizing the coordinated development of land and sea [5,23]. Although many studies have carried out strategic research on the development and utilization of the Beibu Gulf in Guangxi, such as the economy, environmental protection, topography and the human–land relationship of the coastal zone [13,24], the overall development and utilization of the Beibu Gulf coastal area and the coordinated development of land and sea are not clear. Moreover, the planning and regulation of coastal zones in response to the contradiction between development and protection are even less so [25].

Therefore, this paper firstly generated land-use maps from 2000, 2010 and 2020 by interpreting the remote sensing images, then analyzed the spatial and temporal evolution of the territorial pattern of the Beibu Gulf from 2000 to 2020. Lastly, we proposed a comprehensive carrying capacity evaluation system and discussed the spatial function zones of the coastal area of the Beibu Gulf. Our study could provide a scientific basis for regional development and territorial space planning in the coastal area of Beibu Gulf. The scientific questions of this paper are: (1) How has the territorial pattern of the coastal areas of the Beibu Gulf changed in the past 20 years? (2) How can we carry out the comprehensive carrying capacity evaluation of the Beibu Gulf coastal area? (3) How can we reasonably carry out spatial functional zoning and functional management and control under the existing comprehensive carrying capacity of the country?

## 2. Materials and Methods

### 2.1. The Study Area

**Figure 1:**
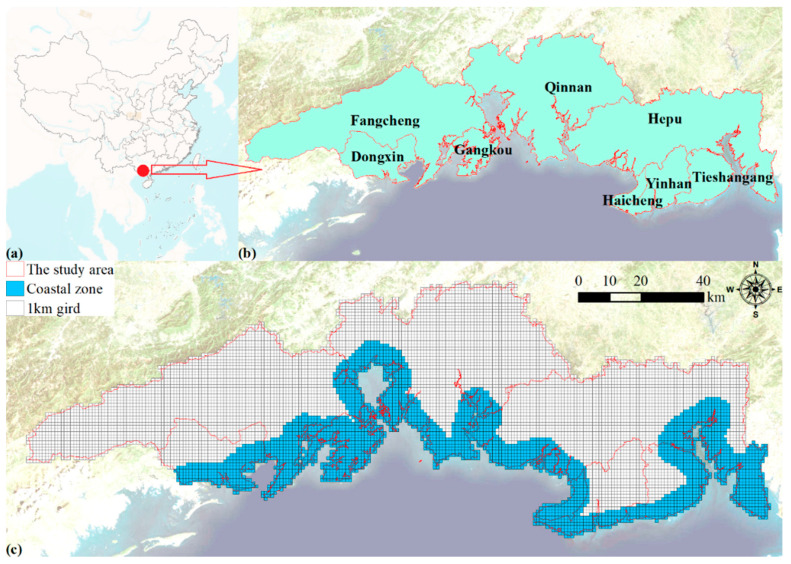
The geographical location (**a**), 8 counties (**b**), 1 km grid and coastal zone (**c**) in the study area.

Beibu Gulf is located in the southern part of Guangxi, bordering Guangdong from the Ximi River estuary in the east (Figure 1a). The coastline of Beibu Gulf is about 1600 km, and it is surrounded by Hainan Island. Its annual average temperature is 20–26 °C. The coldest months are January and February, with temperatures ranging from 15.5–21 °C, while the hottest month is 27–29 °C in July. The average annual sunshine time is 1750–2650 h. In general, the Beibu Gulf area is full of light, and is warm all year round, with evergreen seasons. It is a natural semi-enclosed harbor with a good marine ecological environment, and has a unique location advantage, a new north–south land route connecting the western region of my country and the Indo–China Peninsula. The Beibu Gulf port group plays a role in connecting domestic and foreign transportation and promotes the intersection of the 21st Century Maritime Silk Road and the Silk Road Economic Belt.

We selected 8 districts and counties along the coast of Beibu Gulf (Figure 1b). Its total area is about 8840 km, including 3 districts and 1 county in Beihai City (Haicheng District, Yinhai District, Tieshangang District and Hepu County), 1 district in Qinzhou City (Qinnan District), 3 districts and counties of Fangchenggang City (Gangkou District, Fangcheng District, Dongxing City). The study area is located at the junction of the South China Economic Circle, the Southwest Economic Circle and the ASEAN (Association of Southeast Asian Nations) Economic Circle. As an important bridge and base for China-ASEAN comprehensive cooperation, the study area is the only coastal area and estuary in the western development area, with obvious geographical advantages, superior natural geographical location and prominent strategic position.

### 2.2. Data Sources

First, 30 m spatial resolution remote sensing images were used to interpret land-use maps and identify the territorial space in the study area. Landsat 7 satellite images were collected in 2000, Landsat 5 satellite images were collected in 2010 and Landsat 8 satellite images were collected in 2020. These remote sensing images were downloaded from the geographic information data cloud platform (http://www.gscloud.cn/, accessed on 17 January 2021), and high-quality remote sensing images of the cloud-free, vegetation growing season and non-growing season were selected within the research scope to ensure interpretation. In addition, we obtained the digital elevation data (DEM, Digital Elevation Model) of the study area from this website.

Meanwhile, we carried out field surveys through GPS. The survey contents include land-use identification of random sampling points interpreted from remote sensing data, survey of forest plots and distribution, survey of main crop types and growth cycles, urban social and economic development and marine economic development. Digital maps obtained from the National Geographic Information Center of China (http://www.webmap.cn/, accessed on 17 January 2021) and the Resource and Environment Data Cloud Platform (http://www.resdc.cn, accessed on 17 January 2021), including street-level maps, administrative division demarcation line, urban trunk road network, railway network, rail transit station and river system maps, which were used to help with remote sensing interpretation and land-use feature recognition.

### 2.3. Territorial Pattern Analysis

#### 2.3.1. Land-Use Classification

Remote sensing image processing includes image preprocessing, sample selection, classification, post-classification processing and accuracy verification. Since there are very few grasslands in the Beibu Gulf, we constructed the land-use classification system (Table 1), which includes 7 types of land use: farmland, orchard, aquaculture land, forestland, wetland, bare land and built-up land. Meanwhile, we classified these land-use types into three territorial spaces.

This paper carried out sample selection from the bottom up; that is, each type was divided into subclasses according to the spectral characteristics, and the sample selection accuracy was ensured by the degree of sample separation, and then they were combined after classification. The samples all have obvious spectral information and a certain area (Table 2), and the separation degree of each sample is >90%. The image classification adopts the comprehensive classification method of supervised classification and expert knowledge judgment. The expert knowledge is mainly combined with the data of land-use status map, water resources, road traffic network map/multi-level road network map and other data to set and adjust.

Meanwhile, we used the random point method, which is a completely unconstrained method of laying points, to identify the classification accuracy. The main principle of this method is to divide the area under study into equal-sized grids and place a random sampling point within each grid. In this study, 100 points were randomly generated for each land-use type, and then the GPS was used to verify the consistency between the decoded and real terrain. The classification accuracy is 85.63% in 2000, 81.27% in 2010 and 89.96% in 2020, respectively, which meets the study’s needs.

#### 2.3.2. Land-Use Change

(1) Land-use structure information entropy

Land-use structure information entropy (Equation (1)) can be used to describe the change degree of land-use structure in the study area.

(1) $$H=-\overset{n}{\underset{i=1}{\sum }}A_{i}*lnA_{i}$$

where *H* represents the land-use information structure entropy and *A_i_* represents the area proportion of the *i*-th land-use type to the total area of the study area (dimensionless).

(2) Land-use dynamic degree

The land-use dynamic degree (Equation (2)) refers to the average rate of quantitative change of a certain land-use type in a period, which can describe the structure intensity of land changes in this period, and the higher the value, the more severe the change of this type.

(2) $$K=\frac{A_{m}-A_{n}}{A_{m}}\times \frac{1}{T}\times 100\%$$

where *K* represents the land-use dynamic degree in the period from *m* to *n*. *A_m_* and *A_n_* represent the land-use area o in *m* and *n* years, respectively. *T* is the difference of *m* and *n*.

(3) Landscape pattern index

Territorial pattern is made up of land-use patches of different sizes and shapes, and the spatial distribution characteristics of these land-use patches can effectively reflect the spatial pattern. In this study, 10 landscape pattern indexes (Table 3) were selected from the two scales (class and landscape scales) to analyze the distribution and change characteristics of a territorial pattern.

#### 2.3.3. Hierarchical Clustering Analysis

Hierarchical Clustering Analysis is a technique used to find the underlying structure or clustering tendency of objects through an iterative process that associates (agglomerative methods) or dissociates (divisive methods) the objects based on the information contained in the fingerprint matrix [26,27]. In this paper, the hierarchical agglomerative clustering method was used to cluster the future land use of different counties according to the similarity of the objects. Compared to other clustering methods, the agglomerative methods provide structured clustering with valuable information on the levels of similarity and relative distance between clusters [28]. Additionally, the Euclidean distance was used to calculate the similarity value between the territorial pattern of each county, which was used as a criterion to construct the clustering tree [26].

### 2.4. Comprehensive Carrying Capacity Evaluation

**Figure 2:**
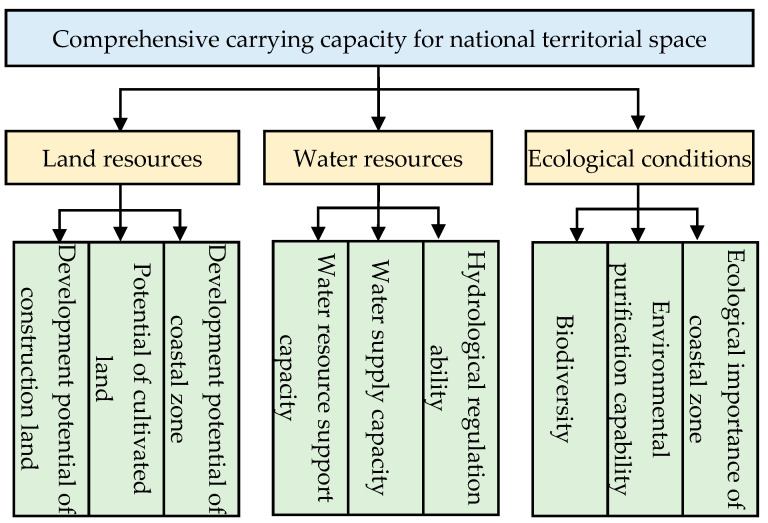
Evaluation index system of comprehensive carrying capacity of national territorial space.

A comprehensive carrying capacity evaluation index system (Figure 2) was estimated by following the principles of science, difference, hierarchy, dynamics and feasibility [29]. The evaluation index system has three levels: the target layer, the criterion layer and the indicator layer [30]. The criterion layer includes three dimensions of land resources, water resources and ecological conditions, and each criterion corresponds to three indicators.

Moreover, the 1 km grid is used as the basic evaluation unit (Figure 1c), and the study area is divided into 9675 grids to carry out the bearing capacity evaluation. The coastal zone is in the cross-coupling and interaction zone of the marine and land environments, and we set the area where the coastline buffers 5 km to the land as the coastal zone, involving a total of 3039 grids to evaluate the development potential of the coastal zone and the ecological importance of the coastal zone.

#### 2.4.1. Carrying Capacity Index Calculation

(1) Development potential of construction land

According to the land-use plan, topography and other conditions, the construction and development suitability was evaluated and classified into grades. The two types of evaluation results of the total area of most suitable development *E*1 and the total area of suitable development *E*2 were compared with the current construction land. The total area of the study area was regarded as the maximum development and construction activities in this area. Based on this, the limit development intensity index (Equation (3)) and the current development intensity index (Equation (4)) were calculated, and then we calculated the development potential of construction land in this area (Equation (5)).

(3) $$LDI=\left[\left(E1+E2\right)\cup^{\;}N\right]/S$$

(4) DI=N/S

(5) p=1−DI/LDI

where *LDI* and *DI* are the ultimate development intensity index of construction land and the current development intensity index, respectively; *p* is the development potential of construction land; *E*1 and *E*2 are the total areas for most suitable development and suitable development; *N* is the total area of the current construction land; *S* is the total area of the study area.

(2) Potential of cultivated land

According to the cultivated land protection red line, existing cultivated land distribution, topography, human disturbance and other conditions, the cultivated land suitability was evaluated. The ratio of the suitable cultivated land area to the total area of the study area is regarded as the largest potential for cultivation activities in the region, and then the current situation is eliminated. The potential of cultivated land was calculated by Equation (6).

(6) c=(E−F)/S

where *c* is the potential of cultivated land; *E* is the total area of suitable cultivated land; *F* is the current scale of cultivated land; *S* is the total area of the study area.

(3) Development potential of coastal zone

The development potential of the coastal zone was calculated by the weighted summation of the area of each functional zone and the influence coefficient of the coastal zone (Equation (7)), which can not only reflect the intensity of marine development and utilization in a region, but can also reflect the potential growth of marine output in the future.

(7) $$R=\sum_{i=1}^{n}h_{i}\times a_{i}/S$$

where *a_i_* is the total area of the *i*-class marine functional area; *h_i_* is the influence coefficient of the *i*-class marine functional area; *n* is the total number of types of marine functional areas; *S* is the total area of the evaluation unit.

(4) Water resource support capacity

Referring to related studies [31], the Modified Normalized Difference Water Body Index (MNDWI) at the pixel scale was calculated using Equation (8) to represent the water resource support capacity, and then the average regional water resource support capacity was calculated according to the MNDWI of each pixel and the number of pixels in the region (Equation (9)).

(8) $$MNDWI_{i}=(Green_{i}-MIR_{i})/(Green_{i}+MIR_{i})$$

(9) $$W=\sum_{i=1}^{n}MNDWI_{i}/n$$

where *MNDWI_i_* and *W* are the *i*-th pixel and the regional water resource support capacity, respectively; *Green_i_* and *MIR_i_* are the green light band and mid-infrared band of pixel *i*; *n* is the total number of pixels in the 1 km grid.

(5) Water supply capacity

Firstly, the water supply capacity of each pixel was calculated according to the water supply service value per unit pixel and the maximum water supply service value in the region (Equation (10)), and then the water supply capacity of each grid was calculated by Equation (11).

(10) $$WC_{i}=WP_{i}/WP_{max}$$

(11) $$WC=\sum_{i=1}^{n}WC_{i}/n$$

where *WC_i_* is the water supply capacity of the *i*-th pixel; *WP_i_* is the water supply service value of the *i*-th pixel; *WP_max_* is the largest water supply service value in the region; *WC* is the average water supply capacity of the region; *n* is the total number of pixels in the 1 km grid.

(6) Hydrological regulation ability

Firstly, the hydrological regulation ability of each pixel was calculated according to the hydrological regulation service value of unit pixel and the maximum hydrological regulation service value in the area (Equation (12)), and then the hydrological regulation ability of each grid was calculated by Equation (13).

(12) $$H_{i}=WR_{i}/WR_{max}$$

(13) $$H=\sum_{i=1}^{n}H_{i}/n$$

where *H_i_* is the hydrological regulation capacity of the *i*-th pixel; *WR_i_* is the hydrological regulation service value of the *i*-th pixel; *WR_max_* is the largest hydrological regulation service value in the region; *H* is the average capacity of regional hydrological regulation ability; *n* is the total number of pixels in the 1 km grid.

(7) Biodiversity

Firstly, the biodiversity level of each pixel was calculated according to the biodiversity value of the unit pixel and the maximum biodiversity value in the area (Equation (14)), and then the biodiversity level of each grid was calculated by Equation (15).

(14) $$BIO_{i}=B_{i}/B_{max}$$

(15) $$BIO=\sum_{i=1}^{n}BIO_{i}/n$$

where *BIO_i_* is the biodiversity level of the *i*-th pixel; *B_i_* is the biodiversity value of the *i*-th pixel; *B_max_* is the maximum biodiversity value in the region; *BIO* is the average biodiversity level in this region; *n* is the total number of pixels in the 1 km grid.

(8) Environmental purification capability

Firstly, the environmental purification capacity of each pixel was calculated according to the environmental purification service value of the unit pixel and the maximum environmental purification service value in the area (Equation (16)), and then the environmental purification capacity of each grid was calculated by Equation (17).

(16) $$EC_{i}=EP_{i}/EP_{max}$$

(17) $$EC=\sum_{i=1}^{n}EC_{i}/n$$

where *EC_i_* is the environmental purification capacity of the *i*-th pixel; *EP_i_* is the environmental purification service value of the *i*-th pixel; *EP_max_* is the maximum environmental purification service value in this region; *EC* is the average environmental purification capacity of the region; *n* is the total number of pixels in the 1 km grid.

(9) Ecological importance of coastal zone

The ecological importance of the coastal zone is calculated according to the existing mangrove area and the total potential mangrove area (Equation (18)), which can reflect the vulnerability of mangroves in each district and county and the status of marine ecological protection.

(18) $$SEA=S/S_{max}$$

where *SEA* is the ecological importance of the coastal zone, *S* is the mangrove area in 2020, and *S_max_* is the maximum mangrove area of historical mangroves.

#### 2.4.2. Weight of Carrying Capacity Index

The entropy method is used to determine the index weight according to the size of the information provided by the value of each index, which can avoid errors caused by human factors [32], thus profoundly reflecting the utility entropy value of the index information. In the comprehensive evaluation, the information entropy represents the relative change speed of the index by describing the change rate of the sample data and the relative magnitude of the change in the index value [33]. The index weight (Table 4) determined by the entropy method can fully reflect the pressure level required by the comprehensive carrying capacity and the coordination of the pressure level, which has higher reliability and accuracy than the subjective weighting method. In the criterion layer, the weights of the three dimensions of land resources, water resources and ecological conditions are basically the same, indicating that they are all indispensable in the evaluation of the comprehensive carrying capacity under the overall planning of land and sea.

## 3. Results

### 3.1. Land-Use Change

The main land-use types in the study area were farmland, orchard and forestland (Table 5), and the largest territorial space was the production space. In the past 20 years, the land use changed in stages. From 2000 to 2010, forestland increased at a rate of 26.12%, followed by built-up land at a rate of 153.81%. During this period, the demand for urban development and the high profits of aquaculture may be the main reasons for the changes in the two land-use types. From 2010 to 2020, only the built-up land and wetland increased. The reduction ratio of forestland and orchard was relatively small, while the reduction ratio of farmland was relatively large. In addition, bare land and aquaculture land decreased with the rate of −25.29% and −19.84%, respectively. Overall, farmland changed notably from 2000 to 2020, followed by built-up land. It can be found that built-up land increased by 386.71% from 2000 to 2020, demonstrating that urban development was the most worthy of attention in the period.

Land-use structure information entropy of the study area in different periods were calculated and presented an increasing trend. The structure information entropy was 1.47 in 2000, 1.59 in 2010 and 1.61 in 2020, respectively. Meanwhile, the land-use dynamic degree (Table 6) showed that the change rate of built-up land was larger in the first stage (2000–2010) than in the second stage (2010–2020). It was as high as 25.78% during the entire study period (2000–2020), indicating that economic demands related to human production activities have led to the continuous expansion of cities. The change rate of farmland was similar to that of built-up land, but it showed a decreasing trend throughout the period, and the dynamic degree was much smaller than that of built-up land, which showed the serious transformation of farmland into other land-use types. Overall, the structural information entropy has changed significantly during the study period, and frequent and drastic land-use changes mainly occurred in 2000–2010.

### 3.2. Spatial and Temporal Variation

**Figure 3:**
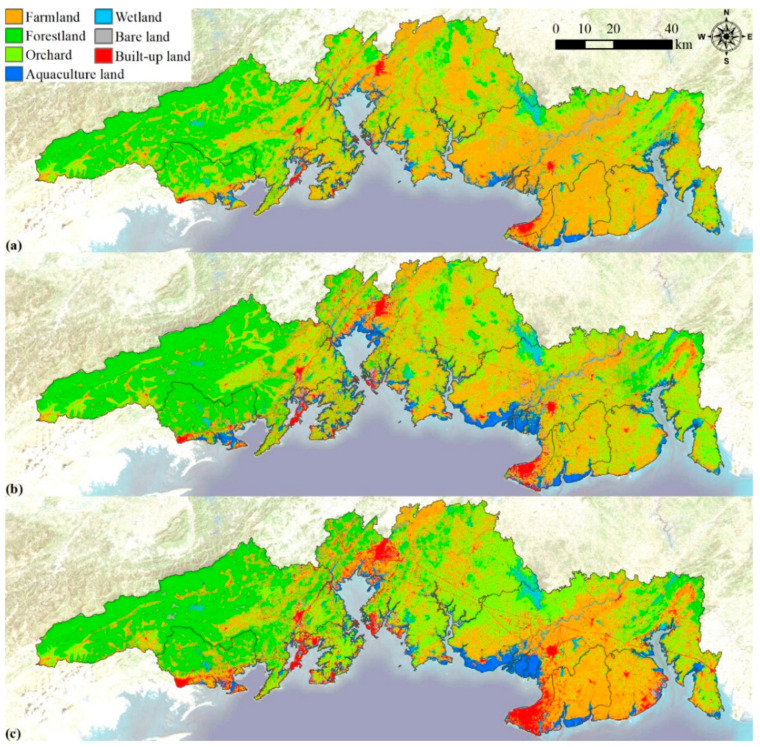
The territorial spaces and land-use pattern in 2000 (**a**), 2010 (**b**) and 2020 (**c**).

There was a significant spatial heterogeneity for territorial pattern in the study area (Figure 3). Forestlands were mainly distributed in the west, farmlands were mainly distributed in the east, wetlands were mainly distributed in the south and orchards were embedded in the whole area. From 2000 to 2010, the increased built-up land was mainly distributed in the coastal areas, the increased forestland was mainly distributed in the west and the increased orchards were mainly distributed in the east. From 2010 to 2020, the increased built-up land was mainly distributed in the coastal areas and inland districts and counties, the increased forestland was small and scattered and the increased orchards were mainly concentrated in the central part. From 2000 to 2020, the increased built-up land was large and widely distributed. They were relatively concentrated for increased forestland in the east and west directions of the study area, and the increased orchards were mainly distributed in the central part. Therefore, urban development led to a continuous increase in built-up land, while ecological protection led to an increase in forestland.

### 3.3. Landscape Pattern Index Change

The landscape pattern indexes at the class scale (Table 7) were calculated by the Fragstat 4.2 software (This free program was originally designed by University of Massachusetts Amherst, Amherst, MA, USA). The CA index was relatively small for the built-up land, but was relatively large for farmland, forestland and orchards, indicating that they were the main landscape patches in this area. NP and PD can reflect the fragmentation degree of various land-use types, and the change trends in NP and PD of various land-use types were almost identical. Although the NP of built-up land was small in 2000, it continued to increase during 2000–2020, and its PD also increased, indicating that cities were expanding explosively. LPI can reflect the landscape dominance degree, and farmland was the dominant landscape in 2000, but forestland was the dominant landscape in 2020. The PAFRAC of each land-use type was not significantly different, and their changes were small, indicating that human activities have not seriously changed the patch shape of each land-use type. IJI can be used to reflect the degree of dispersion and juxtaposition of various landscapes, and the IJI of aquaculture land was the largest, indicating its scattered distribution characteristics. The IJI of forestland showed a continuous increasing trend, indicating that the forestland was relatively concentrated. 

According to the landscape pattern analysis at the landscape scale (Table 8), the territorial pattern in the study area has undergone tremendous changes from 2000 to 2020. The changing PD trend showed that the total landscape fragmentation degree increased first and then decreased under the influence of human activities, and the fragmentation degree in 2020 was greater than that in 2000. The difference in LSI in 2000 and 2010 was small, while the LSI in 2010–2020 was greatly reduced, indicating that the continuous development and utilization of land by humans made the landscape shape of the study area simpler and simpler. The changing CONTAG trend showed that the landscape spread decreased and then increased, but the difference between 2010 and 2020 was small. The COHESION continued to decrease with a small change range, indicating that although human activities had changed the landscape and patch size, the agglomeration degree of the entire landscape did not change significantly. From 2000 to 2020, SHDI first increased and then remained unchanged, indicating that the landscape diversity gradually increased in the process of urban development.

### 3.4. Carrying Capacity Index Evaluation

**Figure 4:**
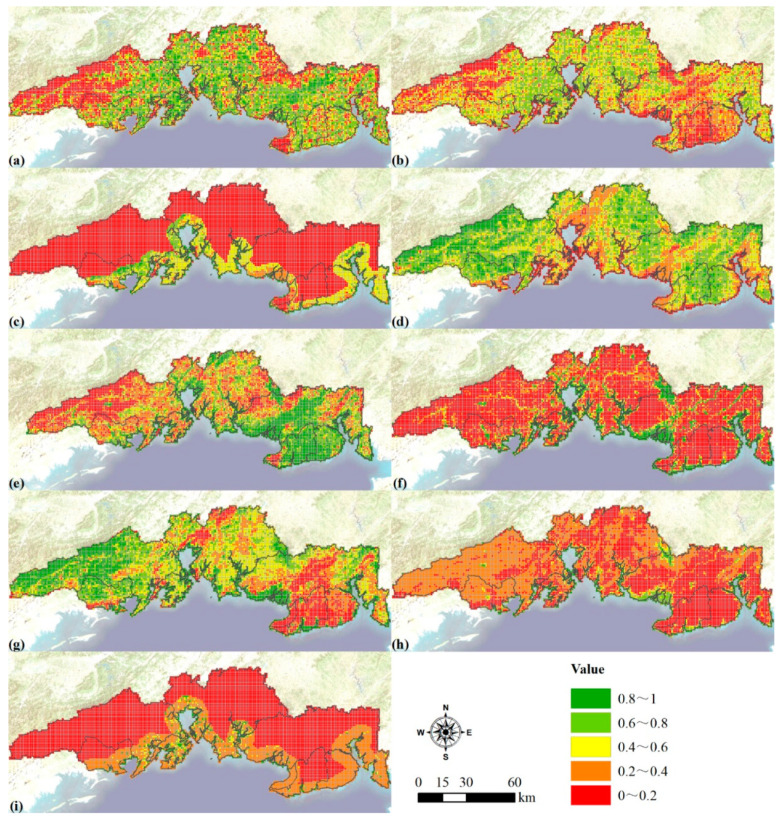
Assessing results for development potential of construction land (**a**), potential of cultivated land (**b**), development potential of coastal zone (**c**), water resources support capacity (**d**), water supply capacity (**e**), hydrological regulation ability (**f**), biodiversity (**g**), environmental purification capability (**h**) and ecological importance of coastal zone (**i**) in the study area with 1 km grid.

We evaluated nine carrying capacity indexes and mapped them to Figure 4. We estimated the development suitability of built-up land. The most suitable area was about 1129.64 km^2^ (13.45%), and the suitable area was about 4614.19 km^2^ (54.80%). Then, the development potential of construction land was evaluated (Figure 4a). We divided the cultivated land suitability evaluation results into three categories: most suitable, basically suitable and unsuitable. Among them, the most suitable area was about 2647.08 km^2^ (29.94%), and the basic suitable area was about 3738.85 km^2^ (42.29%). The potential of cultivated land then was evaluated (Figure 4b), which was an important basis for ensuring food security. The development potential of coastal zone in the southern region was higher than that in the western, northern and central areas among 3039 grids (Figure 4c).

The MNDWI of the study area was calculated based on the Landsat data in 2020, and then the water resource support capacity within the 1 km grid was calculated (Figure 4d). The west, northeast and east of Yinhai District and the west of Tieshangang District showed strong water resource support capacity, while the water resource support capacity was lower in the southern part of Yinhai District and the southeastern part of Tieshangang District. The water supply capacity showed significant spatial heterogeneity (Figure 4e). On the whole, the water supply capacity of the western part was the strongest, followed by the central part, and it was the weakest in the eastern part. The hydrological regulation capacity ranged from strong to weak in the order of western, central and eastern (Figure 4f). Among them, the hydrological regulation ability of the western part of Fangcheng District, the northern part of Dongxing City, the northeastern part and the southwestern part of Hepu County was stronger than other regions.

The biodiversity level in the western and central parts was higher than that in the east (Figure 4g). Among them, the biodiversity levels in the southwest of Hepu County, the southeast of Qinnan District, the adjacent areas of the northwest of Hepu County, and a small part of the southern part of Yinhai District were significantly higher than other areas. The environmental purification capacity was at a lower middle level, and the western region was stronger than the central and eastern regions (Figure 4h). The ecological importance of the coastal zone was calculated based on the existing mangroves in 2020 and the potential mangroves (Figure 4i). The ecological importance of the central part of the port area and the southern coast of Qinnan area was medium, and even a small part of the area was above the medium level. The rest of the southern coastal area was mainly characterized by weak ecological importance.

### 3.5. Comprehensive Carrying Capacity

**Figure 5:**
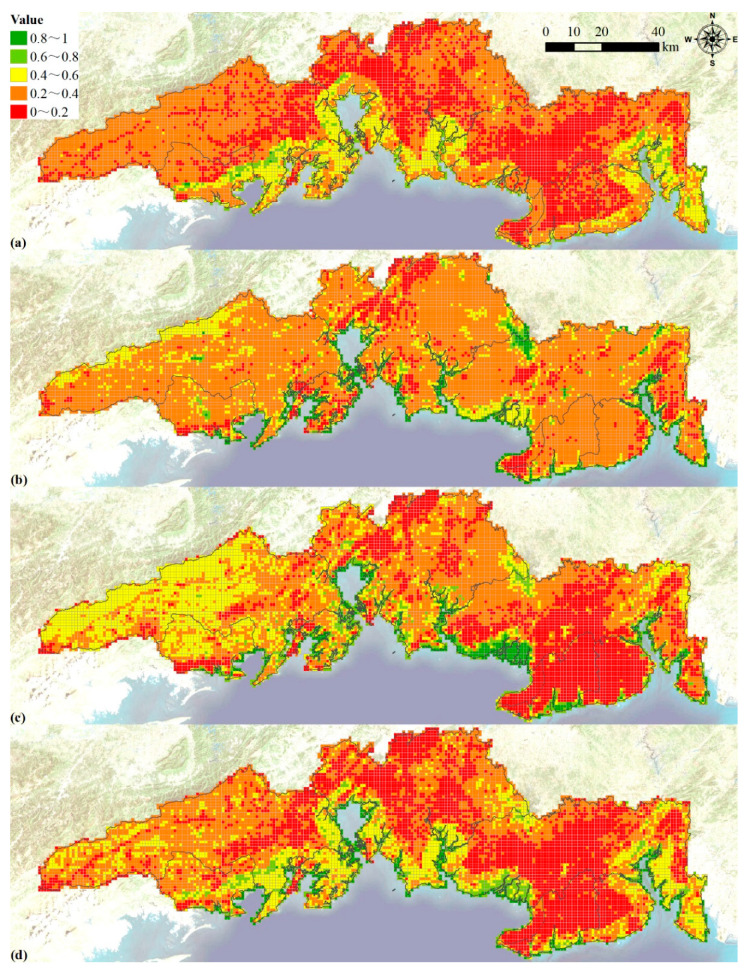
Assessing results for land resources (**a**), water resources (**b**), ecological conditions (**c**) and comprehensive carrying capacity (**d**) in the study area with 1 km grid.

The three criterion layers were assessed through the gray correlation degree (Figure 5). The comprehensive level of land resources was higher in the southern part than the northern part (Figure 5a). The comprehensive capacity of land resources was moderate in Fangcheng District, southern Qinnan District, and southeastern Hepu County, while it was low in the eastern Fangcheng District, Qinnan District, Hepu County, Haicheng District, Yinhai District and Tieshangang District. The comprehensive level of water resources was high in the western and northeastern parts (Figure 5b). Water resources support capacity, water supply capacity and hydrological regulation ability were higher in the western and northeastern parts than other areas. In addition, the comprehensive level of water resources was low in the central and southeastern parts. The comprehensive level of ecological conditions was high in the west part (Figure 5c), which was generally consistent with the spatial pattern of biodiversity level. The comprehensive level of ecological conditions was higher in the southwest of Hepu County and the southern part of the Yinhai District, which was combined by the biodiversity level and the environmental purification capacity. It was moderate in the western part, which is dominated by forest land, while it was poor in the eastern part, which was dominated by farmland, where human activities were relatively strong.

Combined with the assessment results of land resources, water resources and ecological conditions, the comprehensive carrying capacity was high in the south and low in the north, and high in the west and low in the east (Figure 5d). The comprehensive carrying capacity around coastal areas was at a moderate level, except for in the Haicheng District. Although the water resource level in the western and northeastern parts was relatively high, their land resource and ecological condition levels were relatively low, so that the comprehensive carrying capacity was relatively low.

## 4. Discussion

### 4.1. Dynamics of Territorial Spaces in the Past 20 Years

(1) Land-use changes

The largest territorial space was the production space, and farmland, forestland and orchard were the main land-use types in the study area (Table 5). Though on-the-spot investigation, we learned that the farmland was mainly represented by paddy fields, and the orchards were mainly represented by dwarfed lychee, longan, passion fruit and other economic forests. Meanwhile, the forestland was mainly represented by evergreen broad-leaved forests. From 2000 to 2020, the dominant territorial space was the production space, but the dominant landscape changed from farmland to orchard. In 2000, the total area of farmland was relatively high, accounting for about 41.6%, followed by orchards, accounting for about 28.9%. The third landscape was forestland, accounting for about 17.3%. In 2010, farmland was still the main landscape in the region, accounting for 31.2%, followed by orchards, accounting for 30.8%. In 2020, orchards became the main landscape, accounting for about 30.8%, followed by farmland, accounting for about 29.8%. Therefore, the production space of farmland and orchard dominated the landscape base of this area at different times. By comparing and analyzing the variations among these land-use types, farmland and orchard accounted for a relatively high proportion, and the agricultural economy in this region may be dominated by primary industries such as agricultural and fruit [5]. In addition, although the proportion of forestland and built-up land was small, their distribution range was wide and continuously increasing during 2000–2020, indicating that this area has also paid more attention to ecological protection and urban development in the past two decades [8,15,34].

(2) Spatial and temporal variations

Built-up land, farmland, forestland and orchard accounted for a large proportion of the study area (Table 5). The increases in these land-use types were affected by regional socio-economic conditions and ecological processes [3,5], and can reflect the social, economic and ecological changes in the study area in different periods (Table 6). At the same time, the landscapes varied significantly among the eight districts and counties because of the differences of geographical conditions, social economy and ecological environments (Figure 3). In general, forestland was mainly distributed in the west, farmland was mainly distributed in the east and orchards were embedded in the whole area (Figure 3). According to the cluster analysis, the eight districts and counties could be divided into three typical landscapes: ① The urban landscape represented by Haicheng District. Their main land-use types are farmland and built-up land. Over the past 20 years, the built-up land increased and has become the main landscape base in these areas. ② The typical agricultural landscape represented by Hepu County, Qinnan District, Gangkou District, Yinhai District and Tieshan Port District. Their main landscape bases were farmlands. ③ Agricultural and forestry landscape represented by Dongxing City and Fangcheng District. Their main landscape was forestland, followed by orchard and farmland.

(3) Landscape pattern index changes

The landscape pattern changes among these land-use types, which have their own characteristics. At the class scale, the built-up land presented an explosive expansion, but it preferred a relatively scattered construction to a big pie expansion. This expansion style made the fragmentation more and more serious, and the fragmentation degree of the wetland first decreased and then increased, but the changing trend was not obvious. The fragmentation degree of farmland and forestland increased first and then decreased. Meanwhile, the patch number of farmland decreased in a sporadic state, while the patch number of forestland increased in a sporadic state. The orchard changed into pieces, and the new patches made the orchard larger plaque, thereby reducing its fragmentation degree. In addition, human activities made the landscape shape of the study area simpler. The spread degree first decreased and then increased, and human activities changed in landscape and patch sizes.

### 4.2. Zoning Layout and Control Points Based on Comprehensive Carrying Capacity

**Figure 6:**
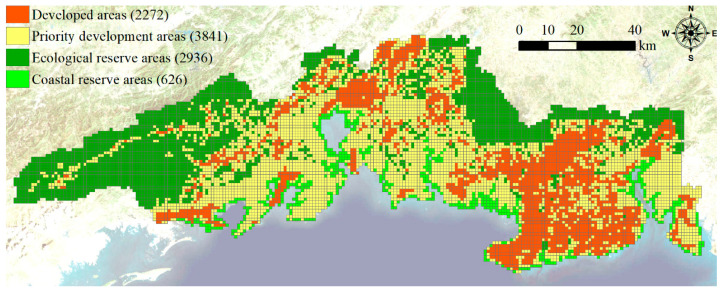
Zoning layout based on comprehensive carrying capacity.

Comprehensively considering the land and sea planning, main functional zoning, the actual natural resources, ecological environments, social and economic developments and the comprehensive carrying capacity evaluation results, we divided the study area into four categories: developed areas, priority development areas, ecological reserve areas and coastal reserve areas (Figure 6).

(1) Developed areas

The developed areas are the urban lands (living space) and permanent basic farmlands (production space) which have been developed and constructed at present, indicating the planning, and built-up areas are mainly for the service industry and for living. According to the zoning layout map (Figure 6), the developed areas are mainly distributed in the Haicheng District, Yinhai District, Tieshangang District and Hepu County, the northwest of Qinnan District, the south of Dongxing City and the southwest of the Gangkou District. Large-scale construction activities are prohibited in these core areas, mainly to maintain the current production and living functions. In the non-core areas, urban construction and development could be appropriately carried out to promote industrial production and the quality of life of residents. This could also be achieved by planting fruits and vegetables to improve agricultural production (Table 9). In general, the developed areas accounted for about 23% of the study area.

(2) Priority development areas

The priority development areas mainly referred to the existing unused areas with high development and utilization intensity or with huge development potential suitable for development and utilization, including the existing urban construction area, port and port-side industrial area and planned construction land. They are mainly distributed in the Gangkou District, the east of Dongxing City and the Fangcheng District, Qinnan District, the west and east of Hepu County and the north of Tieshangang District (Figure 6). These areas accounted for about 40% of the study area. For the priority development areas, the constructed points are to increase fishery production and output through aquaculture, port construction and port-to-port transportation, to accelerate the development of port-related industries, and to encourage the development of the area with high intellectual factor density, to promote the development of the tourism industry (Table 9). In addition, regarding the development and construction of the urban area, ecological protection should be paid attention for a good ecological environment.

(3) Ecological reserve areas

Ecological reserve areas refer to natural ecological spaces that play an important role in conserving water sources, maintaining soil and water, regulating floods, preventing wind and sand fixing and maintaining biodiversity. These areas include nature reserves, national geological parks, forest parks, marine parks and other ecologically sensitive areas, accounting for about 30% of the study area. Ecological reserve areas were mainly distributed in the northwest of the Fangcheng District and Dongxing City, the eastern part of Qinnan District and the northern part of Hepu County (Figure 6). It is necessary to strictly control the urban construction and human activities, such as large-scale land reclamation. The main control points were to protect and restore natural vegetations, implement major ecological restoration projects, and appropriately carry out eco-tourism to promote the development of the tourism industry (Table 9).

(4) Coastal reserve areas

The main functions of coastal reserve areas were to protect marine and coastal resources, including wetlands, mangroves and coastal protection forests distributed in the northern part (Figure 6). The coastal reserve areas accounted for about 7% of the study area. Coastal reserve areas should be subject to mandatory protection, focusing on ecological restoration of mangroves, reforesting mangroves in suitable restoration areas and carrying out tending and upgrading in degraded areas, thereby improving the quality and function of mangrove ecosystems (Table 9). Though the ecological restoration, the coastal tourism industry could be appropriately developed to improve the regional economic level.

### 4.3. Limitations and Uncertainties

Some limitations and uncertainties in this paper should be acknowledged. First, various operations such as acquisition, processing and analysis of remote sensing images would introduce uncertainty. In terms of remote sensing image classification, the classification results may be misclassified and omitted due to the limitation of the spatial resolution of remote sensing image itself, which leads to low classification accuracy [35,36]. To improve the image classification accuracy, this paper adopted a comprehensive classification method with supervised classification and expert knowledge judgment. Additionally, we also further validated the classification results by conducting a field survey through GPS. However, these methods only minimize the uncertainty generated by remote sensing image processing; they cannot eliminate it. Second, this paper used landscape pattern indexes to analyze the response of landscape patterns in the region, but the pattern characteristics indicated by it are often incomplete, and each has its own limitations [37,38]. Different indexes have different sensitivities to spatial magnitude and granularity, which will exhibit significant scale effects [37]. The scale effect significantly affects the landscape pattern index, which is an important manifestation of its uncertainty and is regarded as an unavoidable “natural error” in landscape pattern analysis. Finally, the study of comprehensive carrying capacity lacks a scientific and complete research system, and it has not yet constructed a universal evaluation index system [39,40], so the obtained results have certain limitations. In the future, it is necessary to synthesize the interrelationships and influence mechanisms among various elements and conduct different models’ integration and comprehensive research in order to build a comprehensive and universal carrying capacity evaluation system.

## 5. Conclusions

In the past 20 years, human activities have significantly changed the territorial spatial pattern of the study area. Among them, built-up land and forestland presented an increasing trend. Changes in regional economic development demands have made aquaculture land and orchards increase first and then decrease, while farmland and wetland are seriously damaged. Farmland, orchard and forestland accounted for more than 80% of the total area, and the spatial heterogeneity was significant for these land-use types. From the perspective of horizontal spatial distribution, forestland is mainly distributed in the west, farmland is mainly distributed in the east, wetland is mainly distributed in the south and orchards are embedded in the whole area. Meanwhile, we constructed a comprehensive carrying capacity evaluation index system. The carrying capacities were assessed from the three dimensions of land resources, water resources and ecological conditions; the comprehensive carrying capacity was high in the south and low in the north, and high in the west and low in the east. Taking into account the actual situation of natural resources, ecological conditions, social and economic development, protection and development in the coastal area, spatial functional zoning was proposed under the existing comprehensive carrying capacity. We divided the study area into four categories: developed areas, priority development areas, ecological reserve areas and coastal reserve areas. The territorial space development zoning and control points provided the basis for future territorial development and territorial space control and optimization.

## Figures and Tables

**Table 1:** Classification of territorial space and land-use type.

Territorial Space	Land-Use Type	Description
Production space	Farmland	Agricultural production land
	Orchard	Fruit production land
	Aquaculture land	Aquaculture production land
Ecological space	Forestland	Forests with the crown density more than 0.2
	Wetland	Oceans, rivers, lakes and mud flats
	Bare land	Abandoned land and bare rock
Living space	Built-up land	Towns, roads and settlements

**Table 2:** Land-use types and classification examples in remote sensing images for three territorial spaces.

Territorial Space	Classification Examples in Satellite Images			
Production space	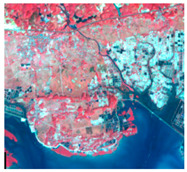	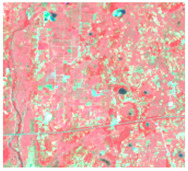	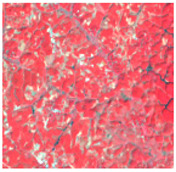	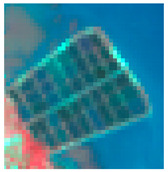
	Farmland (Paddy field)	Farmland (Dry land)	Orchard	Aquaculture land
Ecological space	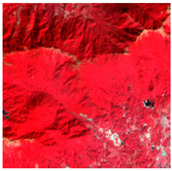	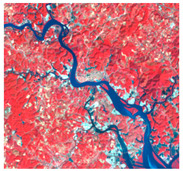	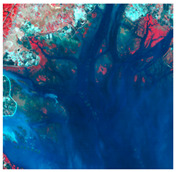	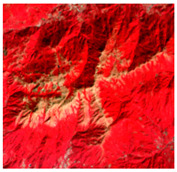
	Forestland	River	Lake	Bare rock
Living space	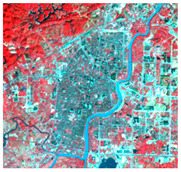		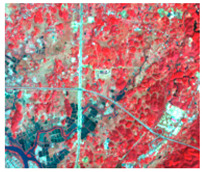	
	Town and settlement		Road	

**Table 3:** Landscape pattern index in our study.

Landscape Pattern Index	Study Scale	Significance
CA (Class Area)	Class scale	Describing the differences in patch distribution of different land-use types
NP (Number of Patches)	Class scale	Describing the number of patches of different land-use types and their degree of fragmentation
PD (Patch Density)	Class and landscape scales	Describing the fragmentation degree of patches of different land-use types for class scale, and the average fragmentation degree of all land-use patches in the entire study area for landscape scale
LPI (Largest Patch Index)	Class scale	Describing the dominant land-use type
PAFRC (Perimeter Area of Fractal Dimension)	Class scale	Describing the shape characteristics of patches of different land-use types
IJI (Interspersion and Juxtaposition Index)	Class scale	Describing the spatial distribution and juxtaposition of patches of different land-use types
LSI (Landscape Shape Index)	Landscape scale	Describing the comprehensive shape characteristics of patches of different land-use types throughout the study area
CONTAG (Contagion Index)	Landscape scale	Describing the extension trend of patches of different land-use types in the whole study area
COHESION (Cohesion index)	Landscape scale	Describing the degree of aggregation of patches of different land-use types throughout the study area
SHDI (Shannon’s Diversity Index)	Landscape scale	Describing each land-use type that tends to be distributed evenly throughout the study area

**Table 4:** Weights of each indicator for comprehensive carrying capacity.

Target Layer	Criterion Layer	Weight	Indicator Layer	Weight
Comprehensive carrying capacity	Land resources	0.399	Development potential of construction land	0.10374
			Potential of cultivated land	0.08778
			Development potential of coastal zone	0.20748
	Water resources	0.229	Water resources support capacity	0.08015
			Water supply capacity	0.0916
			Hydrological regulation ability	0.05725
	Ecological conditions	0.372	Biodiversity	0.17856
			Environmental purification capability	0.08184
			Ecological importance of coastal zone	0.1116

**Table 5:** Land-use area and their changes during 2000 and 2020 in the study area.

Territorial Space	Land-Use Type	Land-Use Area (%)			Change Rate of Land-Use Area (%)		
		2000	2010	2020	2000–2010	2010–2020	2000–2020
Production space	Farmland	41.61	31.23	29.82	−24.95	−4.51	−28.33
	Orchard	28.88	30.82	30.76	6.7	−0.18	6.51
	Aquaculture land	2.82	4.43	3.55	57.21	−19.84	26.02
Ecological space	Forestland	17.25	21.76	21.66	26.12	−0.45	25.56
	Wetland	3.28	3.39	4.12	3.08	21.74	25.5
	Bare land	3.86	4.27	3.19	10.65	−25.29	−17.34
Living space	Built-up land	1.38	3.51	6.74	153.81	91.76	386.71

**Table 6:** Land-use dynamic degree during 2000 and 2020 in the study area.

Territorial Space	Land-Use Type	Land-Use Dynamic Degree (%)		
		2000–2010	2010–2020	2000–2020
Production space	Farmland	−3.56	−0.56	−1.89
	Orchard	0.96	−0.02	0.43
	Aquaculture land	8.17	−2.48	1.73
Ecological space	Forestland	3.73	−0.06	1.7
	Wetland	0.44	2.72	1.7
	Bare land	1.52	−3.16	−1.16
Living space	Built-up land	21.97	11.47	25.78

**Table 7:** Landscape pattern indexes for class scale in the study area.

Territorial Space	Land-Use Type	Time	Landscape Pattern Index					
			CA	NP	PD	LPI	PAFRAC	IJI
Production space	Farmland	2000	368,541.18	47,276	2.34	4.77	1.48	55.29
		2010	276,307.74	58,652	2.9	1.29	1.51	60.24
		2020	264,707.73	55,799	2.76	3.08	1.47	65.5
	Orchard	2000	255,871.71	73,713	3.64	1.07	1.49	49.86
		2010	273,161.07	65,598	3.24	1.08	1.49	49.07
		2020	272,339.91	50,350	2.49	2.72	1.46	61.48
	Aquaculture land	2000	32,269.5	9991	0.49	0.08	1.51	75.77
		2010	47,184.66	5822	0.29	0.62	1.48	90.26
		2020	35,061.84	13,763	0.68	0.31	1.49	74.6
Ecological space	Forestland	2000	152,552.52	17,630	0.87	3.48	1.39	35.16
		2010	192,423.33	30,192	1.49	6.42	1.45	54.39
		2020	191,535.3	17,938	0.89	5.38	1.4	56.42
	Wetland	2000	168,618.69	10,992	0.54	7.16	1.41	79.39
		2010	165,802.41	11,485	0.57	6.79	1.43	90.76
		2020	171,593.19	22,319	1.1	2.87	1.43	90.92
	Bare land	2000	34,228.71	45,739	2.26	0.01	1.45	44.91
		2010	38,382.57	37,661	1.86	0.02	1.36	66.4
		2020	30,995.55	29,072	1.44	0.04	1.39	74.2
Living space	Built-up land	2000	12,466.44	8322	0.41	0.1	1.43	72.21
		2010	32,659.29	25,807	1.27	0.19	1.44	80.2
		2020	63,821.79	44,004	2.17	0.44	1.45	75.27

**Table 8:** Landscape pattern indexes for landscape scale in the study area.

Time	PD	LSI	CONTAG	COHESION	SHDI
2000	11.69	216.46	46.14	99.60	1.62
2010	12.34	216.24	42.83	99.35	1.73
2020	11.77	191.17	43.40	99.31	1.73

**Table 9:** Zoning layout and control guidance for the study area.

Zone	Main Function	Control Guidance
Developed areas	Production and living	No construction activities and maintaining current functions
Priority development areas	Industrial production and residential life	Urban construction and development
	Agricultural production	Agriculture
	Fishery production	Aquaculture
	Port industry port construction	Port transportation
	Tourism	Modern service industry
Ecological reserve areas	Environmental protection	Ecological protection, ecological restoration, controlled development and some consideration of ecological tourism
Coastal reserve areas	Ecological restoration	Mangrove restoration, coastal tourism

## Data Availability

Not applicable.
